# Delayed plastic responses to anodal tDCS in older adults

**DOI:** 10.3389/fnagi.2014.00115

**Published:** 2014-06-06

**Authors:** Hakuei Fujiyama, Jane Hyde, Mark R. Hinder, Seok-Jin Kim, Graeme H. McCormack, James C. Vickers, Jeffery J. Summers

**Affiliations:** ^1^Human Motor Control Laboratory, School of Medicine, University of TasmaniaHobart, TAS, Australia; ^2^Movement Control and Neuroplasticity Research Group, Department of KinesiologyKU Leuven, Leuven, Belgium; ^3^Motor Behavior Laboratory, Department of Physical Education, Seoul National UniversitySeoul, South Korea; ^4^Wicking Dementia Research and Education Centre, University of TasmaniaHobart, TAS, Australia; ^5^Research Institute for Sport and Exercise Sciences, Liverpool John Moores UniversityLiverpool, UK

**Keywords:** aging, plasticity, transcranial direct current stimulation, transcranial magnetic stimulation, brain-derived neurotrophic factor

## Abstract

Despite the abundance of research reporting the neurophysiological and behavioral effects of transcranial direct current stimulation (tDCS) in healthy young adults and clinical populations, the extent of potential neuroplastic changes induced by tDCS in healthy older adults is not well understood. The present study compared the extent and time course of anodal tDCS-induced plastic changes in primary motor cortex (M1) in young and older adults. Furthermore, as it has been suggested that neuroplasticity and associated learning depends on the brain-derived neurotrophic factor (BDNF) gene polymorphisms, we also assessed the impact of BDNF polymorphism on these effects. Corticospinal excitability was examined using transcranial magnetic stimulation before and following (0, 10, 20, 30 min) anodal tDCS (30 min, 1 mA) or sham in young and older adults. While the overall extent of increases in corticospinal excitability induced by anodal tDCS did not vary reliably between young and older adults, older adults exhibited a *delayed* response; the largest increase in corticospinal excitability occurred 30 min following stimulation for older adults, but immediately post-stimulation for the young group. BDNF genotype did not result in significant differences in the observed excitability increases for either age group. The present study suggests that tDCS-induced plastic changes are delayed as a result of healthy aging, but that the overall efficacy of the plasticity mechanism remains unaffected.

## INTRODUCTION

Transcranial direct current stimulation (tDCS) is a non-invasive brain stimulation (NIBS) technique that induces transmembrane neuronal potential and thus influences the level of cortical excitability (see review Nitsche et al., 2008; Zaghi et al., 2010). It is thought that the neuronal changes associated with the persisting effects of tDCS are analogous to activity-dependent synaptic plasticity, i.e., long-term potentiation (LTP) and long-term depression (LTD; Di Lazzaro et al., 2012). There are a number of lines of evidence for the induction of LTP by DC stimulation. Fritsch et al. (2010) showed that the synaptic effects of direct current stimulation which induced LTP in mouse motor cortex (M1) slices is dependent on *N*-methyl-D-aspartate (NMDA) receptor activation. Ranieri et al. (2012) further demonstrated that DC stimulation applied to rat brain slices modulated LTP in a polarity specific manner, i.e., modulation of LTP was increased by anodal and reduced by cathodal DC stimulation. Pharmacological studies have also shown that tDCS after-effects are affected by NMDA receptor antagonist dextromethorphane (Nitsche et al., 2003a; Ranieri et al., 2012). These results strongly indicate that the effects induced by tDCS share similarities with activity-dependent synaptic plasticity, such as LTP and LTD (Di Lazzaro et al., 2012). Furthermore, DC stimulation also modulates LTP induced by other NIBS techniques and interferes with learning and memory processes which are strongly associated with LTP (Kim and Linden, 2007). Thus tDCS is considered to have capacity to induce LTP/LTD-like plasticity.

The application of tDCS over M1 elicits changes in corticospinal excitability in a polarity specific manner: motor evoked potentials (MEPs) evoked by transcranial magnetic stimulation (TMS) are potentiated by anodal tDCS and suppressed by cathodal tDCS (Nitsche and Paulus, 2000). Furthermore, the technique is extremely well tolerated by most individuals and allows an easily applied sham condition to which the participant is readily blinded. Finally, it has been shown that tDCS can be combined with motor and cognitive tasks to facilitate learning.

Despite the abundance of research on the behavioral effects of tDCS on motor function (see review Reis and Fritsch, 2011) showing increased levels of performance in both healthy (Nitsche et al., 2003b; Antal et al., 2004; Vines et al., 2006) and clinical populations, such as stroke patients (Hummel et al., 2005; Kim et al., 2009), only a limited number of studies have investigated the effects of tDCS over M1 in healthy older adults (Hummel et al., 2010; Zimerman et al., 2013). These two studies did not, however, assess the tDCS-induced changes in corticospinal excitability. While the behavioral improvements suggest that the plastic changes within M1 induced by tDCS were substantial enough to elicit behavioral change, it is unknown whether these neural changes varied relative to the changes expected in young adults. Indeed, there is some evidence that older individuals may have reduced neuroplasticity (Fathi et al., 2010), at least when assessed by the paired-associative stimulation (PAS) technique. The overall conclusion that can be drawn is that there is no consensus on the degree to which neuroplasticity is affected by healthy aging (Oliviero, 2010).

Recently, brain-derived neurotrophic factor (BDNF) a neurotrophin within the secretory protein family, has been suggested to play a crucial role in neuroplasticity and associated learning (Pascual-Leone et al., 2011). The most common forms of BDNF polymorphism are Val66Met and Val66Val (Lu, 2003). These BDNF polymorphisms have been shown to differentially modulate human cortical plasticity as a response to training (Kleim et al., 2006), brain stimulation (Cheeran et al., 2008), and motor learning (Fritsch et al., 2010). Thus, the BDNF example suggests that individuals with a certain genetic predisposition may show a different response to interventions that modulate brain plasticity – either in a use-dependent manner (i.e., motor or cognitive training) or induced via NIBS techniques. The relationship between tDCS induced aftereffects and BDNF genotype, however, is still unclear.

Given the current uncertainty as to the effects of aging on neuroplasticity in M1 and its interaction with BDNF genotype, the present study investigated, for the first time, age-related changes in the degree of plasticity following anodal tDCS (facilitatory) and the influence of BDNF gene polymorphisms on neuroplasticity in the aging brain. Recently, large inter-individual variability in response to NIBS techniques has been recognized (Müller-Dahlhaus et al., 2008; Datta et al., 2012; Hamada et al., 2012; López-Alonso et al., 2014; Wiethoff et al., 2014). Of further interest in the present study, therefore, was to determine whether the responsiveness to anodal tDCS is affected by age and explore whether this inter-individual variation is associated with BDNF genotype.

## MATERIALS AND METHODS

### PARTICIPANTS

Forty healthy volunteers, consisting of 20 older adults recruited from the local community (7 males, 13 females, age *M* = 68.3, SD = 7.9 years) and 20 young adults recruited from students at the University of Tasmania (10 males, 10 females, age *M* = 22.7, SD = 3.3 years), participated in the study. Within the older group, participants had similar socio-economic status and were involved in active social activities and/or paid employment. All participants completed at least high school education. The Mini-Mental State Examination (Dick et al., 1984) was used to screen for cognitive deficits in the sample of older adults. All participants scored within the normal range (score ≥ 26) and were free of any neurological, symptomatic cardiovascular disease, diabetes or hypertension. Ethics approval for the study was obtained from the Human Research Ethics (Tasmania) Network and written informed consent was obtained prior to participation in the study. All participants were screened for contra-indications to TMS. We also considered the effect of physical activity on the response to tDCS. Participants completed the International Physical Activity Questionnaire (IPAQ) which assesses the amount of time during the previous seven days spent engaging in a range of physical activities. The IPAQ has been shown to produce reliable and repeatable measures of physical activities in young (Craig et al., 2003; Hagstromer et al., 2006) and older adults (Tomioka et al., 2011).

### BDNF GENOTYPING

Three young and one older participant did not feel comfortable undergoing the characterization of the genotype. The remaining 36 participants gave written informed consent prior to participation in the DNA sampling. ARMS-PCR (Sheikh et al., 2010) was performed to amplify the BDNF gene region containing rs6265. Using the four primers P1 forward CCTACAGTTCCACCAGGTGAGAAGAGTG, P2 (reverse) TCATGGACATGTTTGCAGCATCTAGGTA, P3 (G allele specific) CTGGTCCTCATCCAACAGCTCTTCTATAAC and P4 (A allele specific) ATCATTGGCTGACACTTTCGAACCCA we could distinguish two allele specific amplicons 253 bp (val) and/or 201 bp (met) along with the 401 bp amplicon (entire rs6265 region as internal control). The ARMS-PCR reaction was carried out in a total volume of 12 μl containing 1× REDExtract-N-Amp^TM^ PCR ReadyMix^TM^ (Sigma-Aldrich, USA), 1 μM of each of the four primers (P1, P2, P3, and P4) and 10 ng of genomic DNA. Thermocycling conditions were: denaturation at 94° C for 3 min, 30 cycles of 95° C for 45 s, 62° C for 60 s and 72° C for 60 s, with a final extension at 72° C for 2 min. ARMS-PCR products were then resolved on a 4% agarose gel. The samples could then be classified as Val/Val (253/253 bp), Val/Met (253/201 bp), or Met/Met (201/201 bp) based on the observed banding pattern. All samples should have the rs6265 internal control (401 bp) band present. Every sample was genotyped from at least two independent PCR reactions to ensure fidelity. Participants were divided into two groups according to their genotype, either (i) homozygous for the val allele (Val/Val) or (ii) homozygous and heterozygous for the Met allele (Met/Met, Val/Met), respectively. Participants and examiner were blinded with respect to the genotype at the time of examination.

### TMS PROCEDURE AND EMG RECORDING

TMS was used to compare corticospinal excitability of projections from the cortical representation of the right forearm flexor, flexor carpi radialis (FCR), within the left M1, before (baseline), immediately after and at 10, 20, and 30 min intervals following tDCS (see Transcranial Direct Current Stimulation section below). Single-pulse TMS was applied using a standard figure of eight coil (7 cm diameter of each wing) connected to a Magstim 200 (Magstim Company, Dyfed, UK). The TMS coil was held tangentially over the scalp to induce a posterior–anterior current flow and to optimally elicit MEPs in the right FCR. We chose a forearm flexor muscle rather than an intrinsic hand muscle, as proximal muscles of the upper limb are often the targets of rehabilitation (Carson et al., 2013). EMG surface electrodes (Ag/AgCl) were placed over the right FCR in a belly tendon montage and signals were amplified with a gain of 1000, band pass filtered (10–500 Hz) and sampled at 2000 Hz using a 16-bit AD system (CED 1902, Cambridge, UK). EMG data were fed to disk for offline analysis. At the beginning of each session each individual’s resting motor threshold (rMT) was determined as the lowest intensity that evoked MEPs of greater than 50 μV in at least three out of five consecutive trials for the FCR (e.g., Carroll et al., 2008). The optimal scalp position for induction of MEPs in the right FCR was marked on the participant’s head using a felt tip pen. With both upper limbs relaxed and resting on a pillow in the participant’s lap, baseline MEP recruitment curves were constructed by applying stimuli in steps of 10% between 90% and 160% of rMT. At each intensity, six pulses were delivered with an inter-stimulus interval of 5 s (Carson et al., 2013); thus each recruitment curve took approximately 4 min to collect. For thirty participants, the order of stimulus intensities was randomized; however, due to a technical problem, for the 10 participants (five males), the randomization of intensities was replaced with counterbalanced intensity steps. As no differences between recruitment curves obtained in a ramping (systematically increased stimulus intensity) and random manner have been observed (Pearce et al., 2013), it is unlikely that the changes to recruitment curve acquisition influenced the results obtained.

### TRANSCRANIAL DIRECT CURRENT STIMULATION (tDCS)

The direct current was generated by a battery-driven constant-direct current stimulator (Magstim Company, Dyfed, UK). The current was delivered to the participant through anodal (5 cm × 5 cm) and cathodal (6 cm × 8.5 cm) conductive rubber electrodes that were placed inside pre-saline soaked and gelled sponges with conductive gel. Following the baseline measurement, either anodal stimulation or sham stimulation was applied. The anodal stimulation involved an initial two-second “ramp-up” period during which the current was brought from 0 to 1 mA. The current then remained at 1 mA for 30 min (Lindenberg et al., 2013). The sham stimulation involved the same initial ramp. However, the current was then immediately ramped down to zero over a 30 s period (Nitsche et al., 2008). The participants were blinded to tDCS conditions and were instructed in both sessions that they may feel a mild itching sensation under the electrodes. That is, the participants were led to believe they were receiving tDCS in both sessions (Kessler et al., 2012). For both anodal tDCS and sham conditions the center of the anodal electrode was placed over the FCR representation in the left primary motor cortex, the location of which was identified in the initial setup phase of the experiment, while the cathodal electrode was positioned over the contralateral supraorbital region.

### PROCEDURE

**FIGURE 1:**
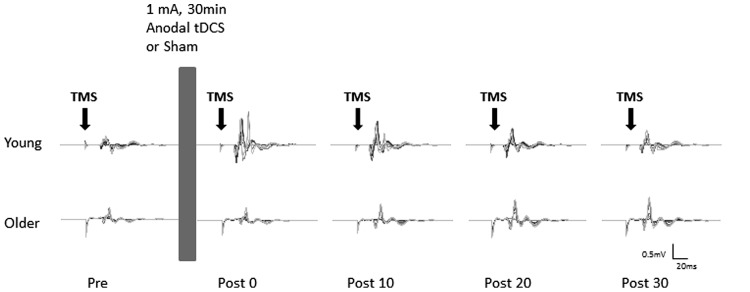
**Example of motor evoked potentials (MEPs) evoked in the right flexor carpi radialis (FCR) at pre and post (0, 10, 20, and 30 min) anodal tDCS from a typical young (top) and older (bottom) participant**. Average MEPs at each stimulus intensity (90–160% of rMT) were overlaid on top of each other.

Participants received sham and anodal tDCS in two separate sessions, one week apart at a similar time of day. The order of sham and anodal tDCS was counterbalanced across participants. During each session the participants were comfortably seated in a chair with their left arm supported and stabilized on a pillow with the elbow in a semi-flexed position to ensure no muscle activations in the forearm and hand muscles. After establishing the rMT, the baseline MEP recruitment curve was recorded. This was followed by 30 min of either sham or anodal tDCS. MEP recruitment curves were then obtained immediately (0 min), 10, 20, and 30 min after cessation of tDCS (**Figure 1**).

### DATA PROCESSING AND ANALYSIS

In presenting the results, the data are expressed as mean (*M*) ± 95% confidence intervals (CI). Using spearman’s bivariate correlations we investigated whether IPAQ score was linearly correlated with normalized area under the recruitment curve (AURC) values. We also considered gender in responses to tDCS, given that there is evidence that PAS-induced neuroplasticity may be reduced in older females (Tecchio et al., 2008). Resting motor thresholds were examined by a 2(AGE: Young, Older) × 2(SEX: male, female) × 2(GENOTYPE: Val66Val, met-carrier) × 2(STIM: anodal tDCS, Sham) repeated measures ANOVA.

MEP amplitude was measured by calculating the peak-to-peak amplitude in a time window 20–100 ms following TMS. MEPs were subsequently averaged across all trials at each stimulation intensity at each time point (pre, post 0, post 10, post 20, post 30) and for each participant. Trials in which root mean square (RMS) EMG exceeded 10 μV (Carson et al., 2004) during the 40 ms immediately preceding the TMS pulse were discarded. The mean MEP amplitude at each intensity and time point was then used to calculate the AURC for each time point. The curve was bounded by TMS intensity using the trapezoidal rule (Potteiger et al., 2002; Carson et al., 2013). More specifically, the following formula was used, $\sum \frac{10\left(a+b\right)}{2}$, where *a* and *b* represent MEP amplitudes at consecutive stimulus intensities, e.g., 90% rMT and 100% rMT. AURC for each post-stimulation time point was then normalized for each participant to AURC obtained at the baseline pre stimulation (anodal or sham) for that participant.

A mixed factorial ANOVA was undertaken to assess post-stimulation normalized AURC (dependent variable) using AGE (Young, Older), SEX (Male, Female), GENOTYPE (Val66Val, met-carrier), STIM (anodal tDCS, Sham), and TIME (post 0, post 10, post 20, post 30) as independent variables. If the sphericity assumption was violated, Greenhouse-Geisser’s degrees of freedom adjustment was applied. Planned contrasts between groups (AGE, SEX, and GENOTYPE) at each time point and across time points for each group separately were conducted to explore significant main and interaction effects.

We performed chi-square tests to evaluate the percentage of responders and non-responders in two groups divided on the basis of BDNF, age, or sex. Non-responders were defined operationally according to the ratio between pre and grand average of the post-anodal tDCS AURC values across time points (post 0, 10, 20, and 30) below 1 (Hamada et al., 2012). We chose this criterion to accept any increases in AURC as a possible neuroplastic response to anodal tDCS.

The critical *p*-value was set at 0.05. Cohen’s *d*, Cramer’s *V* and partial eta squared ($\eta_{p}^{2}$ ) values are provided as measure of effect size, where appropriate. Cut-offs ≥ 0.1 small, ≥0.3 medium, ≥0.5 large were applied for Cramer’s *V*, ≥0.2 small, ≥0.5 medium, ≥0.8 large were applied for Cohen’s *d* and ≥0.01 small, ≥0.06 medium, and ≥0.14 large were applied for $\eta_{p}^{2}$ (Sink and Stroh, 2006).

## RESULTS

Due to a technical error resulting in MEPs not being recorded, data from one young participant was excluded from all analyses. **Table 1** summarizes demographic information, BDNF genotype, and motor thresholds in each session. The BDNF genotype analysis for the remaining 35 participants for whom we had genetic data revealed that 24 were homozygous for the Val allele (Val66Val), while 11 were Met-carriers including seven Val66Met heterozygotes and four homozygous for the Met allele.

**Table 1:** Summary of demographic information and BDNF genotype.

	Age	Sex		BDNF genotype^*^		
	*M* ± *SD*	Male	Female	Val66val	Val66Met	Mat66Met
Young	22.7 ± 3.2	11	9	11	3	2
Older	68.3 ± 7.9	7	13	13	4	2

* Three young and one older participant did not consent to genotype determination.

### RESTING MOTOR THRESHOLD

Resting motor threshold (defined as a percentage of maximum stimulator output) was significantly higher in Met carriers (51.27 ± 3.25%) than Val66Val group (46.48 ± 2.01%), *F*_1,30_ = 4.83, *p* = 0.04, $\eta_{p}^{2}$ = 0.14. rMTs did not vary reliably as a function of age group (Young, 48.37 ± 2.87%; Older, 49.10 ± 2.03%) or session (anodal, 48.63 ± 2.32%; sham, 48.93 ± 2.49%), *F*s < 0.91, *p*s > 0.34, $\eta_{p}^{2}$ = 0.02.

### THE EFFECT OF AGE AND GENOTYPE ON PLASTICITY INDUCED BY ANODAL tDCS

**FIGURE 2:**
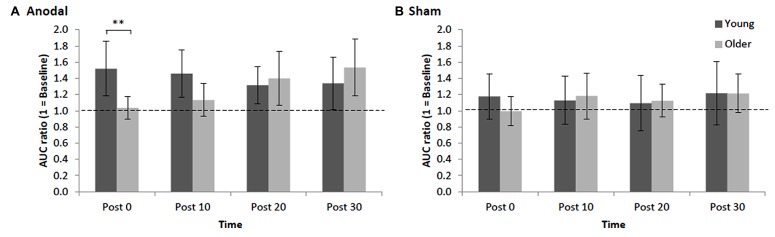
**Mean normalized AURC for young and older adults across post-stimulation [(A) anodal tDCS and (B) sham] time points**. Error bars (95% CI) which include the value 1 (baseline: dotted horizontal line) indicate non-significant differences at that time relative to baseline. Asterisks indicate a significant difference between groups (**p < 0.01).

Repeated measures ANOVA for normalized AURC data revealed that there was a main effect of STIM, *F*_1,3__0_ = 4.97, *p* = 0.03, $\eta_{p}^{2}$ = 0.12, indicating, as expected, that the increase in AURC induced by anodal tDCS (1.34 ± 0.16; +34%, significant increase, *p* < 0.001, *d* = 0.70) was significantly larger than AURC changes following sham tDCS (1.14 ± 0.16). The main effect of AGE was not significant, *F*_1,30_ = 0.11, *p* = 0.74, $\eta_{p}^{2}$ = 0.002, indicating that overall the magnitude of changes in post-tDCS AURC values were not statistically different between young and older adults. However, young and older adults showed different patterns in the modulation of AURC post-tDCS as indicated by a significant interaction of AGE x TIME, *F*_3,90_ = 4.68, *p* = 0.004, $\eta_{p}^{2}$ = 0.11, which is best interpreted with reference to the significant three-way interaction of AGE x STIM x TIME, *F*_3,90_ = 2.84, *p* = 0.04, $\eta_{p}^{2}$ = 0.07. For young adults, although within the anodal condition AURC values were not significantly different between the different time points (*p*s > 0.17, *d*s < 0.32), AURC values at only post 0 and post 10 following tDCS were significantly higher than respective time points in the sham condition (*p*s < 0.008, *d*s > 0.41). A different response pattern was observed in older adults with AURC values at post 20 (1.40 ± 0.33) and post 30 (1.53 ± 0.35) being significantly higher than post 0 (1.04 ± 0.14) and post 10 (1.13 ± 0.20; *p*s < 0.02, *d*s > 0.58) as well as at the respective post-sham time points (*p*s < 0.02, *d*s > 0.34; **Figure 2**). Within the sham condition, AURC values at each time point were not significantly different, *p*s > 0.13, *d*s < 0.22. Finally, an important finding was that the only between-group difference was observed at post 0 where the AURC value in young adults was significantly larger than the value obtained for older adults at that time point (*p* = 0.01, *d* = 0.85; **Figure 2**). Thus, the interaction between AGE and TIME was predominantly driven by older group showing AURC value changes in time-dependent manner (i.e., delayed response to anodal stimulation), whereas the responses in young group were unchanged across time points following stimulation.

Interestingly, all main effects and interactions including SEX and/or BDNF as a factor were not statistically significant, *F*s *<* 1.47, *p*s > 0.23, $\eta_{p}^{2}$ < 0.04, and all were associated with small effect sizes, suggesting that these factors may not be associated with the neuroplastic changes following anodal tDCS.

### INTER-INDIVIDUAL VARIABILITY OF RESPONSES TO ANODAL tDCS

**FIGURE 3:**
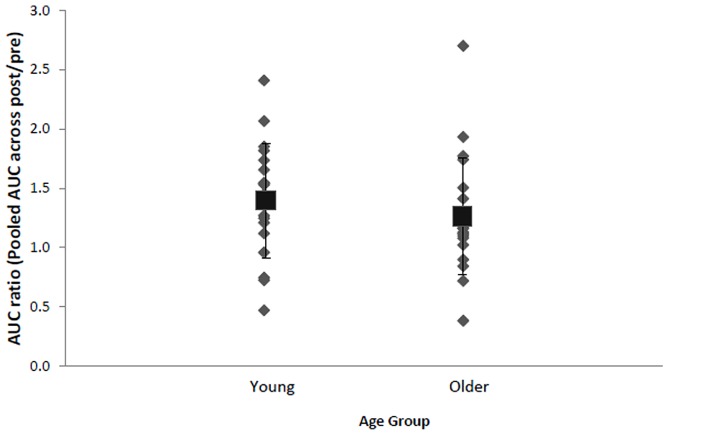
**Mean (black square with error bars denoting SD) and individual AURC ratios (pooled AURC across post-time points divided by baseline pre stimulation) in the anodal tDCS condition for young and older adults**. A ratio larger than 1 indicates AUC increase following anodal tDCS.

Inspection of each individual's data revealed that despite group effects indicating a significant effect of stimulation, there were some individuals who did not display the anticipated increase in AURC following anodal tDCS (**Figure 3**). Approximately 20% of total participants, specifically four young (two males, two females, *M* = 24.5, SD = 3.3 years) and four older females (*M* = 69.0, SD = 9.3 years), were identified as non-responders exhibiting AURC values across post-anodal tDCS time points below 1. Responders consisted of 16 (seven males and nine females) older adults (age range: 60–88 years, *M* = 67.7, SD = 7.8) and 15 young (nine males and six females) participants (age range: 19–29 years, *M* = 23.0, SD = 3.1). Response to anodal tDCS did not differ by BDNF genotype (17 Val66Val and 10 Met-carriers in responders), *χ*^2^(1, *N* = 35) = 1.72, *p* = 0.19, *V* = 0.22, age, *χ*^2^(1, *N* = 39) = 0.01, *p* = 0.94, *V* = 0.01, or sex, *χ*^2^(1, *N* = 39) = 0.82, *p* = 0.37, *V* = 0.15. In non-responders, normalized AURC values between anodal and sham conditions at each time point were not statistically different (*p*s > 0.16, *d*s < 0.41). In both older and younger responders, there were no significant correlations between total IPAQ score and AURC values at any time point following anodal tDCS (*r*s < 0.38, *p*s > 0.16). Physical activity, therefore, did not appear to be associated with corticospinal excitability changes induced by tDCS.

## DISCUSSION

The present study was designed to investigate the age-related changes in the extent of neural plasticity of the corticospinal projections from primary M1 in response to anodal tDCS. For young adults the largest potentiation in excitability was observed immediately following a 30 min application of anodal tDCS. Although AURC values were not significantly different between the post-stimulation time points, only up to 10 min post-anodal tDCS showed significantly higher corticospinal excitability than sham condition. Although this result is inconsistent with a recent finding (Monte-Silva et al., 2013), in which a *reduction* in corticospinal excitability relative to baseline was observed following a 26 min period of anodal stimulation, consistent with the present data is a report of increased corticospinal excitability following a 40 min bout of anodal tDCS (Williams et al., 2010). Although we believe it is extremely unlikely that the single-blinded approach utilized in the current study affected the results, it may be desirable to use double-blinded approach in the future studies to remove the possibility of any confound.

For older adults, in contrast, post-stimulation potentiation was delayed with the largest response occurring 30 min after the stimulation. Indeed, potentiation immediately after stimulation and 10 min thereafter was significantly different to neither sham nor baseline. Importantly, neither BDNF nor sex appeared to influence the neuroplastic changes induced by anodal tDCS. In light of this finding, implications for the use of tDCS in older populations are discussed below.

### DELAYED INCREASE IN CORTICOSPINAL EXCITABILITY FOLLOWING ANODAL tDCS IN OLDER ADULTS

We found that the magnitude of anodal tDCS-induced plasticity in older adults was not significantly reduced (as inferred by way of inferential statistics) relative to young adults. Although the average level of potentiation (averaged over the four post-tDCS time points) was lower in older adults (29% relative to pre-stimulation levels) than younger adults (41%), the difference was not statistically significant. What was most striking, however, was our novel, and potentially important, finding that older adults exhibited a delayed response to anodal tDCS relative to young adults. As seen in **Figure 2**, corticospinal excitability in older adults did not peak until 30 min after cessation of stimulation, whereas young adults exhibited their peak potentiation *immediately* following stimulation. Indeed, at 0 and 10 min after tDCS older adults’ corticospinal excitability was not different from baseline, while young adults showed significant potentiation at these early time points. At 20 and 30 min post-anodal tDCS both groups exhibited significant potentiation relative to baseline.

A number of previous studies have reported a decreased capacity for neuroplastic changes within the motor network with advancing age, both in response to motor training (Sawaki et al., 2003; Rogasch et al., 2009 ) and in response to NIBS techniques including PAS (Fathi et al., 2010) and repetitive TMS (rTMS; Müller-Dahlhaus et al., 2008; Fathi et al., 2010; Todd et al., 2010). While PAS and rTMS, including theta burst stimulation (TBS), protocols are thought to elicit LTP- or LTD-like plasticity by changing NMDA-dependent glutamatergic transmission (Stefan et al., 2000; Wolters et al., 2003; Huang et al., 2005, 2007; Ziemann et al., 2008), tDCS influences resting membrane potentials by affecting sodium and calcium channels or by shifting electrical gradients which influence the electrical balance of ions across the neural membrane (Liebetanz et al., 2002). It could therefore be the case that the delayed, but not diminished, tDCS-induced plastic changes evident in older adults is a result of the stimulation targeting different plasticity mechanisms than those targeted by rTMS, TBS and PAS, where age-related declines in plasticity have been observed. That said, it must be noted that age-related decline in plasticity is *not* a ubiquitous finding, as a number of papers indicate preserved neural plasticity in response to motor training (Cirillo et al., 2010, 2011; Hinder et al., 2011, 2013a, b) or NIBS paradigms (e.g., TBS; Di Lazzaro et al., 2008). These discrepancies between individual studies may perhaps reflect large inter-individual variability in response to training programs or brain stimulation interventions.

### VARIABILITY OF INDIVIDUALS’ RESPONSE TO tDCS

Consistent with recent reports using tDCS (Datta et al., 2012; López-Alonso et al., 2014; Wiethoff et al., 2014), rTMS (Maeda et al., 2000; Hamada et al., 2012; López-Alonso et al., 2014), and PAS (Müller-Dahlhaus et al., 2008; López-Alonso et al., 2014), we observed that a number (8 out of 39, or approximately 20% of participants) of participants did not show the expected corticospinal excitablity increase following anodal tDCS. The aftereffects of rTMS protocols including TBS have also been reported to be highly variable between individuals, irrespective of age (Hamada et al., 2012). As a consequence, some recent studies have separated participants into responders and non-responders (e.g., Müller-Dahlhaus et al., 2008; Hamada et al., 2012). It should be noted that all those participants in the current study who exhibited reductions in excitability following anodal tDCS lie within 2 SDs of the mean response for their age group. As such, given a spread of responses within a normal distribution, it might be that these participants did not necessarily display *atypical* responses. That is, there was not a bimodal distribution in the present data reflecting those individuals who responded to tDCS in the expected manner, and those who showed the opposite effect. We suggest, therefore, that rather than indicating a clear pattern of “responders” and “non-responders,” the present data indicate variability around the expected response.

Recently, it has been hypothesized that the inter-individual variability in response to NIBS may be the result of different populations of neurons being stimulated more easily in different people at different times (Hamada et al., 2012). Variability in the response to TMS may not be due to differences between individuals in the plasticity of cortical synapses, but instead may result from individual differences in the recruitment of cortical neurons (Hamada et al., 2012). Furthermore, recent modeling analysis has shown that the current flowing from anodal tDCS is highly influenced by variations in cortical gyri/sulci, suggesting that the effects may not be homogeneous throughout the stimulated area for an individual and depend on the anatomical characteristics in the individual M1 (Datta et al., 2012). Therefore, the observed variability to anodal stimulation in the present study could, at least in part, be explained by individual differences in the anatomical structure of the brain. In addition, we cannot rule out a possibility that other factors played roles in the inter-individual variability in the response to anodal stimulation. Since, for example, emotional states (Baumgartner et al., 2007; Coombes et al., 2009) and motor activities (Koeneke et al., 2006) modulate corticospinal excitability, it may be useful to apply a questionnaire to investigate emotional state and activities prior to the assessment.

We also examined whether BDNF gene polymorphism affects the efficacy of anodal tDCS to induce plasticity. In the human brain, BDNF is thought to modulate NMDA receptor-dependent LTP and LTD (Antal et al., 2010). Neuronal activity in response to experience and environmental demand appears to enhance the local synthesis/secretion of neurotrophins, which in turn regulates synaptic efficacy or growth (Rossini et al., 2007). In contrast to Antal et al. (2010), we did not find that individuals carrying the Val66Met polymorphism had enhanced anodal tDCS-induced plasticity compared to Val66Val carriers. Rather, our results are consistent with two previous studies showing no difference between BDNF genotypes with regard to corticospinal excitability changes following cathodal tDCS over M1 (Cheeran et al., 2008; Di Lazzaro et al., 2012). In the present study, the only group difference observed was in rMT, with Met carriers showing significantly higher rMT than the Val66Val group. Similarly, we did not find any sex difference in response to anodal tDCS. Although the present sample was larger than previous studies examining the relationship between NIBS induced plasticity and BDNF genotype, studies using much larger sample sizes with a balanced number of participants across the polymorphism are clearly required before a definite conclusion can be drawn regarding the impact of BDNF on neuroplasticity mechanisms.

### POSSIBLE MECHANISMS UNDERLYING DELAYED RESPONSE TO tDCS AND IMPLICATIONS

Although the neurophysiological mechanisms underlying the delayed response to anodal stimulation in older adults are unknown, one possibility is that deterioration at the microstructural level in the aging brain may contribute to the delayed response. During aging there is progressive accumulation of damaged molecules and impaired energy metabolism in brain cells (Yu et al., 2002). The aging process compromises neuroprotective and neurorestorative mechanisms involving glial cells (Yu et al., 2002). Recently, it has been speculated that tDCS may affect glial cells rather than neurons directly (Ruohonen and Karhu, 2012). Therefore, the degradation process with advancing age in glial cells may be associated with the delayed response to anodal stimulation in older adults. The next important question is whether the observed response to anodal tDCS in older adults is as long lasting as the increased corticospinal excitability relative to baseline in young adults which persisted for up to 30 min after the stimulation.

Recent studies investigating the effect of tDCS on motor learning have utilized a “gating” strategy in which the excitability of M1 is transiently increased via DC stimulation concurrent with motor task performance(Ziemann and Siebner, 2008). In gating studies, tDCS is often applied at the initial stage of the learning (e.g., Waters-Metenier et al., 2014). Given that older adults showed delayed potentiation in corticospinal excitability, it may be worth considering the application of stimulation *prior to* motor training in older adults to elicit greatest changes in behavior during motor performance.

In summary, young adults exhibited the largest potentiation in corticospinal excitability immediately after the anodal tDCS, while the potentiation in corticospinal excitability was delayed in older adults. Notably, we failed to observe differences in neuroplastic changes following atDCS by BDNF subtypes and sex, suggesting neuroplastic response following atDCS is possibly independent from these factors. Future work is warranted to investigate the association between neuroplasticity induced by NIBS techniques and skill acquisition in older adults. Further understanding of the neurophysiological mechanisms underlying neuroplasticity in older adults has significant clinical implications for the improving of motor function in aging populations and recovery following stroke.

## Conflict of Interest Statement

The authors declare that the research was conducted in the absence of any commercial or financial relationships that could be construed as a potential conflict of interest.
